# Profile of Arachidonic Acid-Derived Inflammatory Markers and Its Modulation by Nitro-Oleic Acid in an Inherited Model of Amyotrophic Lateral Sclerosis

**DOI:** 10.3389/fnmol.2018.00131

**Published:** 2018-04-30

**Authors:** Andrés Trostchansky, Mauricio Mastrogiovanni, Ernesto Miquel, Sebastián Rodríguez-Bottero, Laura Martínez-Palma, Patricia Cassina, Homero Rubbo

**Affiliations:** ^1^Departamento de Bioquímica, Facultad de Medicina, Universidad de la República, Montevideo, Uruguay; ^2^Center for Free Radical and Biomedical Research, Universidad de la República, Montevideo, Uruguay; ^3^Departamento de Histología y Embriología, Facultad de Medicina, Universidad de la República, Montevideo, Uruguay

**Keywords:** nitro-fatty acid, ALS, neurodegeneration, inflammation, astrocytes, mass spectrometry, lipidomics

## Abstract

The lack of current treatments for amyotrophic lateral sclerosis (ALS) highlights the need of a comprehensive understanding of the biological mechanisms of the disease. A consistent neuropathological feature of ALS is the extensive inflammation around motor neurons and axonal degeneration, evidenced by accumulation of reactive astrocytes and activated microglia. Final products of inflammatory processes may be detected as a screening tool to identify treatment response. Herein, we focus on (a) detection of arachidonic acid (AA) metabolization products by lipoxygenase (LOX) and prostaglandin endoperoxide H synthase in SOD1^G93A^ mice and (b) evaluate its response to the electrophilic nitro-oleic acid (NO_2_-OA). Regarding LOX-derived products, a significant increase in 12-hydroxyeicosatetraenoic acid (12-HETE) levels was detected in SOD1^G93A^ mice both in plasma and brain whereas no changes were observed in age-matched non-Tg mice at the onset of motor symptoms (90 days-old). In addition, 15-hydroxyeicosatetraenoic acid (15-HETE) levels were greater in SOD1^G93A^ brains compared to non-Tg. Prostaglandin levels were also increased at day 90 in plasma from SOD1^G93A^ compared to non-Tg being similar in both types of animals at later stages of the disease. Administration of NO_2_-OA 16 mg/kg, subcutaneously (s/c) three times a week to SOD1^G93A^ female mice, lowered the observed increase in brain 12-HETE levels compared to the non-nitrated fatty acid condition, and modified many others inflammatory markers. In addition, NO_2_-OA significantly improved grip strength and rotarod performance compared to vehicle or OA treated animals. These beneficial effects were associated with increased hemeoxygenase 1 (HO-1) expression in the spinal cord of treated mice co-localized with reactive astrocytes. Furthermore, significant levels of NO_2_-OA were detected in brain and spinal cord from NO_2_-OA -treated mice indicating that nitro-fatty acids (NFA) cross brain–blood barrier and reach the central nervous system to induce neuroprotective actions. In summary, we demonstrate that LOX-derived oxidation products correlate with disease progression. Overall, we are proposing that key inflammatory mediators of AA-derived pathways may be useful as novel footprints of ALS onset and progression as well as NO_2_-OA as a promising therapeutic compound.

## Introduction

Amyotrophic lateral sclerosis (ALS) is a multifactorial disease caused by genetic and non-inheritable components leading to motor neuron (MN) degeneration in the spinal cord, brain stem and primary motor cortex (Al-Chalabi and Hardiman, 2013). ALS appears as a complex syndrome where the defective cellular pathways may not derive solely from a conformational issue, but involve many aspects of cellular physiology. While oxidative stress is increased, neurotrophic support is reduced and glial inflammatory response is oriented toward a harmful side (Rossi et al., 2016). In this regard, transgenic superoxide dismutase (SOD1^G93A^) mice are so far the most widely used model to study ALS. SOD1^G93A^ mutants show a progressive paralytic phenotype caused by degeneration of MNs and exhibit gliosis within the spinal cord, brain stem, and cortex (Philips and Rothstein, 2015). Neuronal degeneration in ALS begins as a focal process that spreads contiguously through the upper and lower MN, implicating an acquired pathogenic mechanism where MN pathology and inflammation actively propagate in the central nervous system (CNS) (Barbeito et al., 2004; Turner et al., 2013). Astrocytes and microglia are the main glial cells involved in immune response of the CNS and pathology associated with these cells is referred as neuroinflammation, now considered a hallmark of ALS (Hooten et al., 2015). In fact, many treatments have been tested on ALS animals with the aim of inhibiting or reducing the pro-inflammatory action of these cells and counteract the progression of the disease. Unfortunately, no therapy that appeared promising in transgenic ALS mice, including many targeting neuroinflammation, has improved clinical outcomes in patients with ALS (Lacomblez et al., 1996a,b; Petrov et al., 2017). Multiple factors provide insight as to why translation of therapeutic benefit from mouse to human has failed. In SOD1^G93A^ transgenic mice, it has been shown that little-to-no effect on overall survival was observed when decreasing or deleting single pro-inflammatory factors such as TNF-α, IL1-β or inducible nitric oxide synthase (NOS2) (reviewed in Hooten et al., 2015). Clearly, the multiplicity of pro-inflammatory cytokines can compensate the absence of any single factor, so far it is unlikely that continuing efforts to target a single factor will provide significant therapeutic benefit in patients with ALS (Cleveland and Rothstein, 2001). Moreover, drugs targeting neuroinflammation such as celecoxib, ceftriaxone, thalidomide, and minocycline were reported to enhance survival in transgenic mice, yet none were effective in human ALS trials. Also, targeting the downstream effect of reactive oxygen species (ROS) has shown benefit in ALS animal models but not in patients; immunosuppressive drugs such as glucocorticoids, cyclophosphamide, azathioprine, and cyclosporine, among others, that have proven efficacy in diverse immunological disorders have not shown efficacy in ALS (reviewed in Hooten et al., 2015). Thus, identifying novel biomarkers can improve the design of novel strategies for early diagnosis and treatment of the disease.

Metabolomic studies search small molecules present in cells, tissues or biological samples, whereas the observation of modifications in these molecules levels in addition to physiological modifications of signaling pathways may aid in elucidating where these changes are occurring, e.g., intracellularly. Blood biomarkers should be used as a tool for monitoring the onset and progression of the disease, the appearance of clinical symptoms as well as the efficiency of the treatment with a drug. A wide range of blood metabolites from <1000 to 1500 Da can be used as potential biomarkers of the disease, in particular those related to fatty acids as arachidonic acid (AA), an abundant unsaturated fatty acid present in brain (Rozen et al., 2005).

Several studies have been performed to determine the role of lipid supplementation and serum lipid profile on ALS onset, progression or fate (Yip et al., 2013; Schmitt et al., 2014; Henriques et al., 2015). Despite the well-known health beneficial effects of ω-3 fatty acids, eicosapentaenoic acid (EPA) supplementation in SOD1^G93A^ mice have shown to increase the progression of the disease shortening the life span when supplemented before clinical symptoms appear (Yip et al., 2013). In addition, an increase on lipid oxidation measured as 4-hydroxynonenal levels was also observed (Parakh et al., 2013); SOD1^G93A^ mice supplemented at the onset of the disease had no effects on animals survival or disease progression (Yip et al., 2013). Of interest, dyslipidemia is a good prognostic factor for ALS patients. In fact, ALS transgenic mice are leaner, hypolipidemic and present a higher metabolic intake of fatty acids in muscle than control animals (Schmitt et al., 2014). Overall, the data in the literature suggest the relevance of fatty acid metabolism changes for the onset and progression of ALS.

Arachidonic acid can be metabolized by the prostaglandin endoperoxide H synthase (PGHS) or lipoxygenase (LOX) pathways being the precursor of a wide variety of anti- or pro-inflammatory compounds such as prostaglandins, leukotrienes, hydroperoxy-(HpETE), or hydroxyl (HETE) derivatives which can be followed in small samples of blood and used as disease biomarkers (Brash, 1999; Rouzer and Marnett, 2005). In fact, ALS mice spinal cord (Hensley et al., 2006) as well as sporadic ALS patients cerebrospinal fluid (CSF) and serum (Almer et al., 2002; Ilzecka, 2003) exhibit increased levels of prostaglandin E_2_ (PGE_2_) (Ilzecka, 2003; Miyagishi et al., 2017). Furthermore, PGHS and PGE synthase-1, which are implicated in PGE_2_ biosynthesis, are significantly increased in the spinal cord of ALS mice (Almer et al., 2001; Miyagishi et al., 2012). The involvement of AA metabolites in ALS was also supported by the increased message and protein levels of 5-lipoxygenase (5-LOX) observed in SOD1^G93A^ mice at 120 days of age (West et al., 2004). Of therapeutic interest, oral administration of the 5-LOX and tyrosine kinase inhibitors nordihydroguaiaretic acid (NDGA), significantly extended lifespan and slowed motor dysfunction in SOD1^G93A^ mice (West et al., 2004). Many of these compounds are able to cross brain–blood barrier (BBB) thus being able to be detected by lipidomic analysis (Wenk, 2010).

Nitro-fatty acids (nitroalkenes, NFA) represent novel endogenously-produced electrophiles that exert potent anti-inflammatory signaling actions (Schopfer et al., 2011). In particular, nitro-oleic acid (NO_2_-OA) is presently de-risked by extensive preclinical toxicology and FDA-approved Phase 1 safety evaluation of synthetic as well as oral formulations being well-tolerated. NO_2_-OA is anticipated to be broader and more efficacious for ALS than those stemming from single target drugs, because of its pleiotropic anti-inflammatory and adaptive signaling actions (Baker et al., 2005; Batthyany et al., 2006; Cole et al., 2007; Freeman et al., 2008; Kelley et al., 2008; Li et al., 2008; Sculptoreanu et al., 2010; Zhang et al., 2010; Artim et al., 2011; Schopfer et al., 2011, 2014). In particular, our team has demonstrated that (1) improving mitochondrial function and reducing oxidative stress at mitochondria prolongs survival in SOD1^G93A^ mice (Miquel et al., 2012, 2014); and (2) NO_2_-OA activates Nrf2-mediated induction of antioxidant defenses in astrocytes that may delay or prevent MN death (Vargas et al., 2005; Diaz-Amarilla et al., 2016). In the present work we analyzed the levels of LOX and PGHS products during disease progression and tested whether NO_2_-OA may delay motor symptoms by its capacity to control secondary neuroinflammation.

## Materials and Methods

### Materials

The 10-nitro-oleic acid isomer (NO_2_-OA) was synthesized as previously described (Woodcock et al., 2006, 2013). 12-hydroxyeicosatetraenoic acid-d_8_ (12-HETE-d_8_), 15-hydroxyeicosatetraenoic acid-d_8_ (15-HETE-d_8_), 5-hydroxyeicosatetraenoic acid-d_8_ (5-HETE-d_8_), prostaglandin D_2_-d_4_ (PGD_2_-d_4_), prostaglandin E_2_-d_4_ (PGe_2_-d_4_), and thromboxane B_2_-d_4_ (TxB_2_-d_4_) were obtained from Cayman Chemicals (Ann Arbor, MI, United States). Oleic acid (OA) was purchased from Nu-Check Prep (Elysian, MN, United States). The solvents used in syntheses were HPLC grade. All other reagents were obtained at the highest purity available from standard supply sources. All other reagents were from Sigma Chemical, Co. (St. Louis, MO, United States) unless otherwise specified.

### ALS Mice

Transgenic mice for the G93A mutation in human SOD1 strain [B6SJL-TgN(SOD1-G93A)1Gur] (Gurney et al., 1994) (Jackson Laboratory; Bar Harbor, ME, United States, SOD1^G93A^) were bred “in house” following international guidelines for ethical animal care and experimentation. Hemizygous SOD1^G93A^ transgenic males were bred with wild-type females from their background strain and the offspring was genotyped with a polymerase chain reaction (PCR) assay on tail snip DNA. Mice housing, handling, sample collection and sacrifice were performed following the guidelines for preclinical animal research in ALS (Ludolph et al., 2010) and in accordance to the protocol approved by the Comisión Honoraria de Experimentación Animal (CHEA), Universidad de la República, Uruguay.

#### Experimental Groups and Treatments

SOD1^G93A^ and non-Tg female mice were divided into different groups to analyze the effects on NO_2_-OA, OA or vehicle administration on AA-derived inflammatory markers and ALS progression. In all cases administration was performed three times a week from day 90 (at disease onset) until end-stage. Onset of disease was scored as the first observation of an abnormal gait or evidence of hindlimb weakness. End-stage of disease was scored as complete paralysis of both hindlimbs and the inability of the animals to right after being turned on a side. Body weight, grip strength (using a grip-strength Meter, San Diego Instruments) and rotarod performance (with a rotarod treadmill Letica ROTA-ROD LE 8200) were measured twice weekly from week 6 on through the completion of the study. The animals were divided in the following experimental groups: (1) SOD1^G93A^ + PEG, *n* = 17; (2) SOD1^G93A^ + OA, *n* = 15; (3) SOD1^G93A^ + NO_2_-OA, *n* = 17; (4) non-Tg + PEG, *n* = 9; (5) non-Tg + OA, *n* = 5; (6) non-Tg + NO_2_-OA, *n* = 10. For some studies, and to minimize the use of animals, groups (5) and (6) were eliminated as they were not statistically different in motor performance from group (4). Grip strength was assessed in almost all animals; for rotarod performance a smaller n from groups 1 (*n* = 10), 2 (*n* = 10), 3 (*n* = 10), and 4 (*n* = 9) was selected. At day 100 (10 days after treatment initiation), 4 animals from group 1, 3 from group 2, 4 from group 3, and 3 from group 4 were processed for histology. Blood samples from groups 1 (*n* = 4), 2 (*n* = 5), 3 (*n* = 5), 4 (*n* = 4), 5 (*n* = 5), and 6 (*n* = 5) were obtained and lipidomic analysis was performed as explained below while these animals sacrificed at end stage and processed for NO_2_-OA quantitation in brains. For all animals, injections were performed avoiding the formation of any lesion at the administration zone.

### Lipidomics

Plasma and brain samples from non-Tg and SOD1^G93A^ mice were obtained at ages (i) 60 days (before disease onset); (ii) 90 days (onset of disease); and (iii) 140 days (end stage, sacrifice) (Ludolph et al., 2010).

Analysis and quantitation of lipids in both plasma and brain were performed by ESI LC–MS/MS. For this purpose, samples were analyzed by direct infusion in a Q-TRAP4500 (ABSciex, Framingham, MA, United States) or coupled to a chromatographic separation in an Agilent 1260 HPLC. For chromatographic purposes, lipids were separated on a RP-C18 column (5 μm, 2 mm × 100 mm, Phenomenex Luna). The elution gradient consisted of solvent A: 0.05% acetic acid and solvent B: acetonitrile, 0.05% acetic acid with the following gradient at a flux of 700 μL/min: 0–0.2 min 30% B; 0.2–10 min 100% B; 10–11 min 100% B; 11–11.1 min 30% B; 11.1–15 min 30% B. The column was maintained during the run at a temperature of 30°C (Morgan et al., 2010; Thomas et al., 2010; Trostchansky et al., 2011; Bonilla et al., 2013). Results were processed using Peak View software (ABSciex, Framingham, MA, United States). ESI-MS/MS was performed using an electrospray voltage set at 5 kV, and capillary temperature of 500°C.

#### Plasma Analysis

A 100 μL blood sample was obtained from each animal, centrifuged at 1500 rpm for 15 min at 4°C and plasma separated. Then, deuterated internal standards were added, lipids extracted using the hexane method as previously reported and analyzed by LC–MS/MS (Trostchansky et al., 2011; Fazzari et al., 2014). Protein content of samples were quantified by using the Bradford method (Bradford, 1976).

#### Brain Analysis

Following sacrifice and dissection, brains were stored at -80°C until used. Before analysis, tissues were homogenized in a Next Advance bullet blender with bullet size and time of homogenization in accordance to the protocols given by the company. Briefly, 100 mg of brain tissue were placed in 1.5 mL tubes and a volume of buffer that is twice the volume of the sample was added. Then, 0.5 mm zirconium oxide beads were added using a volume of beads equivalent to 1x the volume of the sample. Finally, brain samples were homogenized for 3 min at a speed of 8. The supernatant were separated from the breads and deuterated internal standards were added, lipids extracted, suspended in methanol and analyzed by LC–MS/MS (Trostchansky et al., 2011; Fazzari et al., 2014). The standards used were 5-HETE_d8_, 12-HETE_d8_, 15-HETE_d8_, AA_d8_, TxB_2d4_, PGE_2d4_, PGD_2d4_, 9-HODE_d8_, and 13-HODE_d4_.

### Quantitation of NO_2_-OA in Mice Brain

Both non-Tg and SOD1^G93A^ mice were administered subcutaneously with 16 mg/kg/day NO_2_-OA, OA or vehicle. After a week, animals were sacrificed and brain obtained to determine if the nitroalkene was able to cross the BBB. The tissue was homogenized as previously, lipids extracted and NO_2_-OA as well as its β-oxidation products detection and quantitation was performed as reported (Rudolph et al., 2009). For quantitation purposes [C^13^]_18_NO_2_-OA (*m/z* 344/46) was used as internal standard and LC–MS/MS analysis was done with the MRM transitions for NO_2_-OA (*m/z* 326/46) and NO_2_-SA (*m/z* 328/46) (Rudolph et al., 2009). After homogenization, the supernatant was collected and extracted using the Bligh and Dyer method (Bligh and Dyer, 1959) with dichloromethane instead of chloroform. Dichloromethane fractions were pooled, dried and resuspended in 500 μL hexane/methyl ter-butylether/acetic acid (HBA) (Salvatore et al., 2013; Fazzari et al., 2014). Then, the complex lipids from the tissues samples were separated by solid phase extraction using Aminopropyl Sepack Strata NH2 (55 μm, 70 Å) columns, obtaining a set of fractions to analyze: (1) Cholesteryl esters (CE) in hexane; (2) Triacylglicerides (Tg) in hexane/chloroform/ethyl acetate; (3) Diacylglicerides (DAG) and monoacylglicerides (MAG) in chloroform/isopropanol; (4) Free fatty acids (FFA) in diethyl ether/acetic acid (Salvatore et al., 2013; Fazzari et al., 2014). After drying, fractions 1–3 were resuspended in ethyl acetate/66 μM ammonium acetate while the others in methanol. To analyze esterified NO_2_-OA, Tg, and DAG fractions (75 μL) were dried. Then, 900 μL of 0.5 M phosphate buffer pH 7.4 + 10 μL of sodium cholate 40 mg/mL was added and samples sonicated. Finally, samples were incubated at 37°C for 3 h with 0.4 mg/mL of pancreatic lipase under agitation followed by 30 min with 20 mM HgCl_2_. To avoid artifactual nitration during organic extraction, sulfanilamide, and NaN^15^O_2_ were added to the reaction mixture. Finally, samples were incubated with [C^13^]_18_NO_2_-OA, extracted and resuspended in methanol before analysis by LC–MS/MS. In parallel, a standard curve using [C^13^]_18_NO_2_-OA under the same chromatographic and mass sprectrometry conditions was performed for quantitative purposes (Salvatore et al., 2013; Fazzari et al., 2014).

### Immunofluorescence

SOD1^G93A^ and non-Tg mice (*n* = 3 per group) were exposed to treatments or vehicle as described above. Sample processing was similar as described (Vargas et al., 2005). At 100 days, mice were subjected to deep anesthesia (pentobarbital, 50 mg/kg i.p.) and transcardially perfused with 0.9% saline followed by 4% paraformaldehyde fixative in phosphate buffer saline (PBS; pH = 7.4). The spinal cords were removed and post-fixed in the same fixative for 4 h. Lumbar spinal cords were cryoprotected and 30 μm-thick sections were obtained on a cryostat and collected in PBS for free-floating immunofluorescence. After permeabilization (0.25% Triton X-100 in PBS) and blocking unspecific binding (10% goat serum, 2% BSA, 0.25% Triton X-100 in PBS), sections were incubated with primary antibodies diluted in blocking solution for 48 h at 4°C. The primary antibodies used were mouse monoclonal anti-GFAP (1:800, Sigma) and rabbit polyclonal anti-HO-1 (1:300, Enzo Life Sciences) followed by secondary antibodies Alexa Fluor^488^ conjugated goat anti-mouse and Alexa Fluor^594^ conjugated goat anti-rabbit (Invitrogen; 1.5 μg/mL). Images were obtained using a confocal microscope (Leica TCS SP5 II) and quantified using ImageJ software from NIH. Mean gray density was measured in gray scale images from GFAP immunolabeling, and double-labeled GFAP/HO-1 cells were counted using cell counter plugin in the ventral horn of spinal cord. At least 8 images obtained from non-adjacent (separated by 300 μm) sections from each lumbar spinal cord were quantified.

### Statistical Analysis

Quantitation experiments were done for each animal, and data reported as the mean ± SEM for each group of mice. Statistics analyses were performed using the Primer of Bioestatistics Software (Stanton A. Glantz) or GraphPad PRISM software, version 5.1. Motor performance by rotarod or grip strength assessment of the different treatment groups were compared using two-way RM ANOVA with Tukey post-test. Experiments were repeated at least three times and data reported as the mean ± SEM. Comparison of the means was performed by one-way analysis of variance followed by Bonferroni post-test and pairwise analysis was performed by the Student-Newman-Keuls test. Differences were declared statistically significant if *p* < 0.05.

## Results

### Plasma and Brain Levels of LOX and PGHS Metabolites Are Altered in SOD1^G93A^ Mice

**FIGURE 1:**
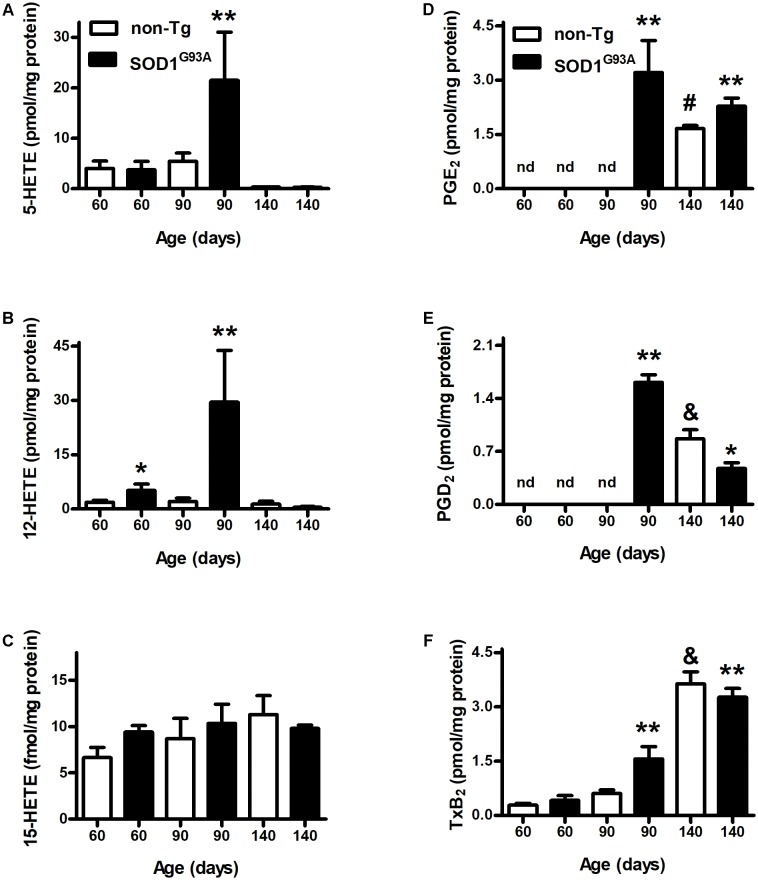
Plasma levels of AA-derived oxidation products. Plasma samples from non-Tg (white bars) and SOD1^G93A^ (black bars) mice were obtained before symptoms (day 60), when clinical symptoms appear (day 90) and at later stages of the disease (day 140). LOX- **(A–C)** and PGHS- **(D–F)** oxidation products were analyzed by LC–MS/MS. Results correspond to the mean ± SEM, with at least six animals per group. ^∗^*p* < 0.05 SOD1^G93A^ mice compared to non-Tg mice at day 60; ^∗∗^*p* < 0.05 SOD1^G93A^ mice compared to non-Tg mice at day 90; and ^#,&^*p* < 0.05 non-Tg mice compared to non-Tg at day 60 or day 90.

**FIGURE 2:**
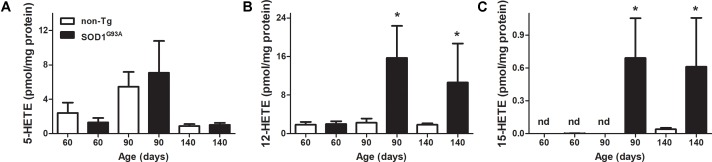
LOX-derived products in SOD1^G93A^ brain through mice life. Brains from non-Tg (white bars) and SOD1^G93A^ (black bars) mice were removed, homogenized, and lipid extracted as explained in section “Materials and Methods.” Then 5-HETE **(A)**, 12-HETE **(B)**, and 15-HETE **(C)** were analyzed by LC–MS/MS. Results correspond to the mean ± SEM, with at least six animals per group. ^∗^*p* < 0.05 SOD1^G93A^ mice compared to non-Tg mice at day 90 and day 140.

We analyzed AA-oxidation products in both plasma (**Figure 1**) and brain (**Figure 2**) before appearance of clinical symptoms (day 60), onset (day 90), and end stage of disease (days 140). When analyzing HETEs, both plasma 5- and 12-HETE levels were greater at the onset of the disease compared to non-Tg mice (**Figures 1A,B**). 12-HETE levels were even higher before clinical symptoms appearance (**Figure 1B**). However, both 5-HETE and 12-HETE showed a huge decrease at day 140 compared to the onset of the disease in SOD1^G93A^ mice returning to pre-symptomatic levels (**Figures 1A,B**). Preliminary data suggest changes in the activity and expression of 5-LOX and 12-LOX during SOD1^G93A^ mice life which may explain the observed results (Trostchansky and Rubbo, unpublished data). In contrast, 15-HETE levels did not show changes between non-Tg and SOD1^G93A^ in any of the analyzed time points (**Figure 1C**). PGE_2_ levels in plasma were higher in SOD1^G93A^ mice at the onset of the disease (**Figure 1D**). An increase in plasma levels was also observed for PGD_2_ and TxB_2_ at same age, suggesting a significant alteration of the AA- PGHS pathway in SOD1^G93A^ mice compared to non-Tg (**Figures 1E,F**). Before symptoms appear, neither PGE_2_ nor PGD_2_ were detected in non-Tg as well as in SOD1^G93A^ mice (**Figures 1D,E**).

LOX- derived products in brains from SOD1^G93A^ mice exhibited a similar behavior of 12-HETE formation through animal’s life (**Figure 2B**): 12-HETE concentration reached its maximum at day 90 (onset of the disease) and maintained until animals were sacrificed in contrast to non-Tg mice where no changes were observed (**Figure 2B**). However, 5-HETE and 15-HETE showed different profiles in brain compared to previously shown plasma data. While plasma 5-HETE increased at the onset of the disease, brain levels did not show any differences between non-Tg and SOD1^G93A^ mice (**Figures 1A**, **2A**). Importantly, and in contrast to that observed in plasma, 15-HETE was not detected before the onset of the disease in SOD1^G93A^ mice (**Figure 2C**).

#### NO_2_-OA Crosses BBB

**FIGURE 3:**
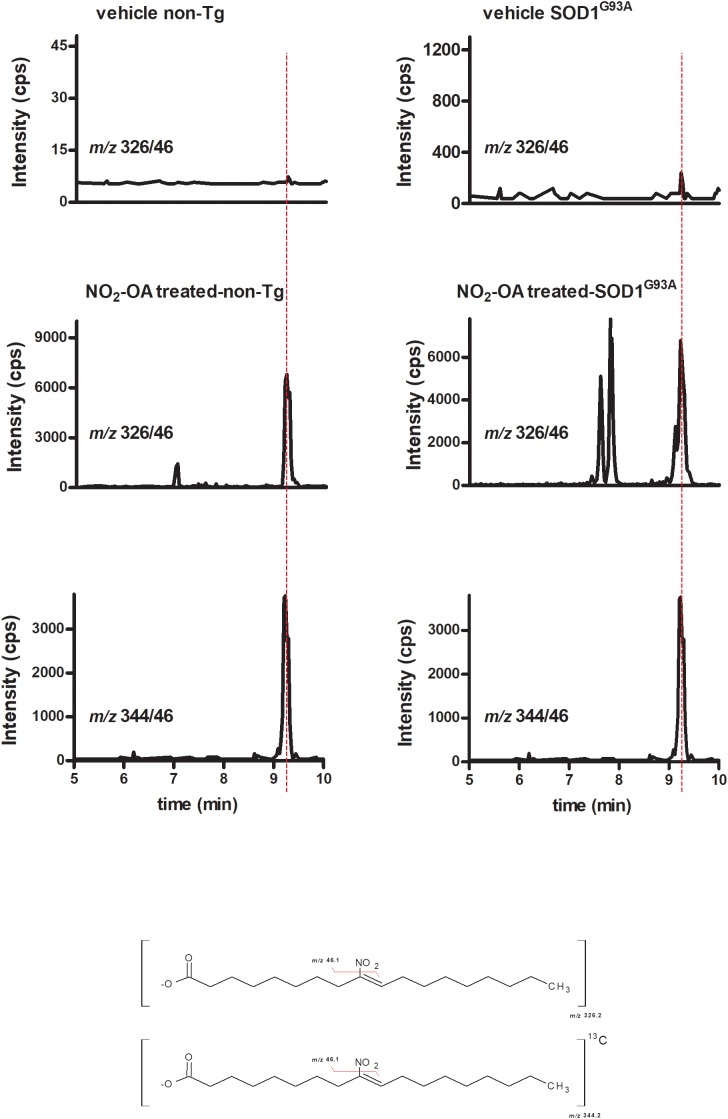
NO_2_-OA crosses BBB in both non-Tg and SOD1^G93A^ transgenic mice. Both non-Tg and SOD1^G93A^ mice were subcutaneously administered with NO_2_-OA (16 mg/kg/day) for a week and brain samples were taken from non-treated animals (top) and treated animals (middle). Brains were homogenized as explained in section “Materials and Methods,” lipid extracted and [C^13^]_18_-NO_2_-OA (lower) added as an internal standard. The presence of NO_2_-OA was followed by the neutral loss of the nitro group by the MRM transition m/z 326/46 for the nitroalkene and m/z 344/46 for the internal standard, as shown at the bottom chemical structures. Retention times in addition to the MRM transitions confirmed the presence of NO_2_-OA in brains due to subcutaneous administration and the levels reached are shown in **Table 1**.

To investigate NO_2_-OA ability to reach the brain, we quantified its concentration as well as its β-oxidation product NO_2_-SA (Rudolph et al., 2009) in brains from animals administered with NO_2_-OA or OA as explained in section “Materials and Methods” (**Table 1**). Nitro-oleic acid was detected in brain from both non-Tg and SOD1^G93A^ mice: **Figure 3** shows the appearance of a product with a MRM transition according to the presence of NO_2_-OA having the same retention time than the internal standard [C_18_]^13^NO_2_-OA. Other key transitions confirmed this result, e.g., the loss of the carboxyl group (data not shown). In both non-Tg and SOD1^G93A^ mice, NO_2_-OA and NO_2_-SA significantly increased when administered subcutaneously, confirming its ability to cross BBB (**Table 1**).

**Table 1:** Determination of NO_2_-OA and NO_2_-SA in brains from Non-Tg and SOD1^G93A^ mice.

	NO_2_-OA (pmol/mg tissue)	NO_2_-SA (pmol/mg tissue)
non-Tg + NO_2_-OA	4.33 ± 1.12	203.11 ± 1.09
SOD1^G93A^+ NO_2_-OA	1.83 ± 0.28	120.71 ± 6.28

#### NO_2_-OA Modulates Brain AA Metabolism

**FIGURE 4:**
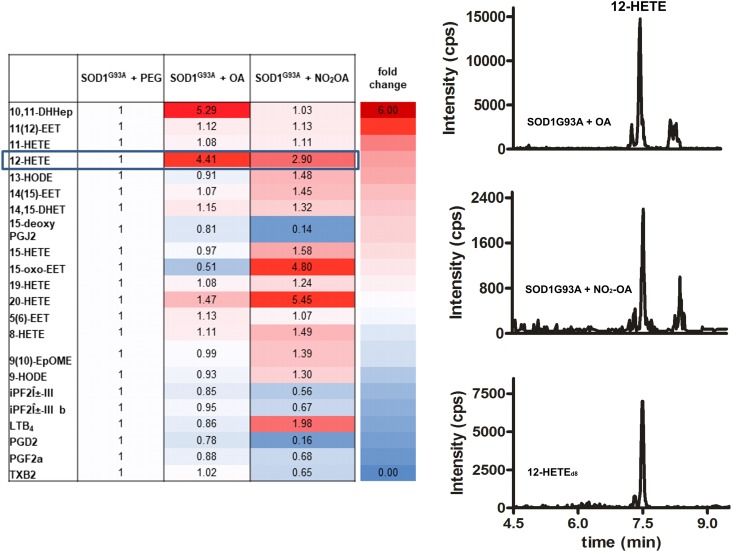
Lipidomic analysis of SOD1^G93A^ mice administered with NO_2_-OA. Lipidomic analysis of brains from SOD1^G93A^ mice obtained after treatment with vehicle (PEG), OA or NO_2_-OA was performed. The table shows fold changes compared to PEG condition for all products. Results are representative of at least three independent experiments (*n* = 9). Color intensities show differences between the groups.

Administration of the nitroalkene exerted changes in the lipidomic profile of SOD1^G93A^ mice compared to controls, with most of the changes being the reduction in the levels of pro-inflammatory and oxidized products (**Figure 4**). Nitro-oleic acid lowered the observed increase in brain 12-HETE levels compared to the non-nitrated fatty acid condition (**Figure 4**). Moreover, NO_2_-OA decreased the production of PGD_2_, PGF_2α_, 15-deoxyPGJ_2_, and TxB_2_ in brains from SOD1^G93A^ mice (**Figure 4**).

#### NO_2_-OA Improves Motor Performance and Neuroinflammatory Markers in SOD1^G93A^ Mice

**FIGURE 5:**
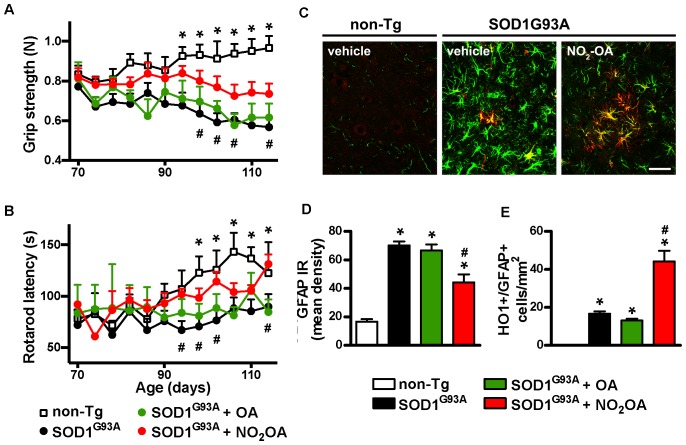
Effects of NO_2_-OA on clinical symptoms and inflammatory markers. Hind limb grip strength **(A)** and rotarod latency **(B)** records from non-Tg or SOD1^G93A^ mice treated with vehicle, OA or NO_2_-OA were obtained. Each data point is the mean ± SEM from 7 to 17 animals per group as indicated in section “Materials and Methods.” ^∗^*p* < 0.05, OA and PEG-treated SOD1^G93A^ compared to non-Tg mice; #*p* < 0.05 NO_2_-OA compared to vehicle or OA-treated SOD1^G93A^ mice. GFAP (green) and HO-1 (red) immunoreactivity in the anterior horn of the spinal cords from non-Tg or SOD1^G93A^ transgenic mice were determined; merged images are shown; HO-1/GFAP double-labeled astrocyte-like cells are observed in yellow; scale bar = 50 μm. **(C)** NO_2_-OA treatment reduces GFAP immunoreactivity **(D)**, and increases the number of HO-1/GFAP immunoreactive astrocytes **(E)** in the ventral horn of the lumbar spinal cord of SOD1^G93A^ mice compared with vehicle or OA-treated animals. Each bar represents the mean ± SEM from at least three animals per group. ^∗^*p* < 0.05 compared to non-Tg mice; #*p* < 0.05 compared to vehicle or OA- SOD1^G93A^ -treated mice.

The final step was to link the capacity of NO_2_-OA to exert beneficial effects in clinical outcome and correlate them with biomarkers of drug action. Motor symptoms, assessed by grip strength and rotarod latency (**Figures 5A,B**) were improved by NO_2_-OA administration. There was no significative difference in motor performance between PEG, OA, and NO_2_-OA treated non-Tg groups in any time point. Astrogliosis, represented by GFAP immunoreactivity, a pathological hallmark of the disease linked to neuroinflammation, was significantly reduced in the spinal cord of SOD1^G93A^ mice, following NO_2_-OA administration compared to vehicle or OA-treated animals (**Figures 5C,D**). In addition, NO_2_-OA induced an increase in HO-1 immunoreactive astrocytes (**Figures 5C,E**).

## Discussion

Neuroinflammation has been reported in both sporadic (sALS) and familiar (fALS), as well as in transgenic models of the disease (reviewed in Barbeito et al., 2004; Hooten et al., 2015; Geloso et al., 2017). Signs of microglia reactivity have been detected before overt symptoms onset, concomitantly with loss of neuromuscular junctions and early MN degeneration. A by product of this process is the production of neurotoxic molecules such as pro-inflammatory cytokines and ROS. These mediators may cause further neuronal damage leading to glial cell activation resulting in a positive feedback loop of neuroinflammation. Due to their high metabolic demand, MNs involved in ALS may be vulnerable to changes in lipid metabolism and fatty acids profile with an abnormal presentation of lipid metabolism (Philips and Rothstein, 2014; D’Ambrosi et al., 2017; Mariosa et al., 2017). Similarly, ALS mice present an increased lipid metabolism being leaner than normal animals displaying an increased uptake of fatty acids in muscles. Importantly, an increase of AA levels and AA-derived inflammatory markers are present in brain during neurodegenerative processes (McNamara et al., 2017). Arachidonic acid can be enzymatically- metabolized to anti-inflammatory or pro-inflammatory products, i.e., PGE_2_ and HETEs (Brash, 1999; Simmons et al., 2004; Haeggstrom and Funk, 2011). It has been reported that PGE_2_ exerts pro-inflammatory action in ALS and other neurodegenerative diseases (reviewed in Cimino et al., 2008), and increases in both serum, CSF and CNS tissues (Almer et al., 2002; Ilzecka, 2003). The observed increase in 12-HETE and prostaglandins in SOD1^G93A^ mice compared to the non-Tg animals suggest that the activity of AA-metabolizing enzymes represent key mediators in the onset and progression of the disease. We have preliminary data showing changes in the expression of both 5-LOX and 12-LOX in brains from SOD1^G93A^ mice compared to non-Tg animals who can explain the observed differences in their enzymatic-derived products concentrations. In addition, both the activity and expression of these AA-metabolizing enzymes in SOD1^G93A^ mice are lower at the end of animal’s life compared to the establishment of the disease age, which can explain the observed decrease in both 5-HETE and 12-HETE levels before mice sacrifice (Trostchansky and Rubbo, unpublished data).

Several work in the literature demonstrate the pluripotent activity of NO_2_-FA, some of them related to NO_2_-OA (Kelley et al., 2008; Liu et al., 2008, 2013; Kansanen et al., 2009; Wang et al., 2010a,b; Sculptoreanu et al., 2010; Artim et al., 2011; Klinke et al., 2014; Zhang et al., 2014; Ambrozova et al., 2016; Koudelka et al., 2016). It has been demonstrated their capacity to modulate inflammatory processes, e.g., induction of HO-1 (Ferreira et al., 2009; Kansanen et al., 2011, 2012; Diaz-Amarilla et al., 2016) or reduction of pro-inflammatory mediators by inhibiting enzyme activities, e.g., inhibition of 5-LOX in neutrophils (Awwad et al., 2014). A recent publication of our group demonstrated that in a cell model of ALS, NO_2_-OA was able to reduce MN death when co-cultured with astrocytes from SOD1^G93A^ mice, in addition to an increase expression of Phase II Antioxidant Enzymes through the Nrf-2 pathway (Diaz-Amarilla et al., 2016). Herein, we demonstrate a protective role of NO_2_-OA in an ALS model due to its ability to cross the BBB and (a) down-modulate PGHS- and LOX-derived inflammatory products and (b) induce HO-1 expression in reactive glia from spinal cord associated to improvement of motor performance. These results further support that up-regulation of ARE/Nrf2 pathway in astrocytes may serve as a therapeutic approach in ALS, as proposed (Vargas et al., 2005).

Our results emphasize that: (1) Changes in prostaglandins and HETEs levels occur at different stages of motor symptoms in SOD1^G93A^ mice; (2) NO_2_-OA was detected and quantitated in CNS as determined by LC–MS/MS after subcutaneous administration; (3) NO_2_-OA administration to SOD1^G93A^ mice reduced prostaglandin and HETEs brain levels. (4) NO_2_-OA significantly delayed grip strength decline and increased rotarod latency compared to vehicle or OA- treated animals and (5) NO_2_-OA reduced astrogliosis as well as increased HO-1 expression in spinal cord of ALS-treated mice.

NO_2_-OA administration was performed when the onset of the disease was established supporting that the nitroalkene may offer benefits during ALS progression. The relevance of our findings, defining the biochemical and physiological responses induced by NO_2_-FAs in ALS, led us to continue to develop a safe and effective treatment using a lipid electrophile-based drug strategy.

## Author Contributions

AT designed and performed the experiments, discussed the results, and wrote the manuscript. MM performed the experiments and discussed the results. EM designed and performed the experiments and revised the manuscript. SR-B designed and performed the experiments. LM-P performed the experiments, discussed the results, and revised the manuscript. PC and HR designed the experiments; wrote and reviewed the manuscript.

## Conflict of Interest Statement

The authors declare that the research was conducted in the absence of any commercial or financial relationships that could be construed as a potential conflict of interest.
